# Leukaemia-associated priapism in children (LAPC): reviewing clinical outcomes and management strategies

**DOI:** 10.3332/ecancer.2025.1860

**Published:** 2025-02-27

**Authors:** Abhijit Shah, KR Surag, Anupam Choudhary, Kasi Viswanath, AVB Krishnakanth, Chaitanya Krishna, Padmaraj Hegde, S Gayathri, PM Swathi

**Affiliations:** 1 Department of Urology, Kasturba Medical College, Manipal, Manipal Academy of Higher Education, Manipal, Karnataka, India; 2 Department of Clinical Hematology, Cellular therapy and Bone marrow transplant, Amrita Institute of Medica Sciences, Kochi, Kerala, India; 3 Department of Pediatric Hemato-Oncology, Kasturba Medical College, Manipal, Manipal Academy of Higher Education, Manipal, Karnataka, India

**Keywords:** leukaemia, priapism, child, outcomes assessment, healthcare, management

## Abstract

**Background:**

Priapism is a prolonged penile erection for more than 4 hours unrelated to sexual stimulation. Rarely, it is the first clinical sign of an underlying haematological malignancy. A similar presentation is noted in childhood leukaemias. Although rare, it is known to occur and, if not managed early, can have poor long-term outcomes in terms of erectile function and psychosexual growth. We present a scoping review of leukaemia-associated priapism in children (LAPC).

**Methodology:**

We researched literature using PubMed, Google Scholar, Embase, Scopus and Cochrane databases from January 1990 to 2024. Applicable search limiters were applied, and grey literature was excluded.

**Results:**

A total of 31 articles were finally included in the review, from which 51 cases of LAPC were isolated and studied. The average age was 11.5 years, with chronic myeloid leukaemia (CML) being the most common malignancy (68.9%), and more than 71% of cases of CML with priapism were detected in the chronic phase. Twenty cases (39.2%) were managed with corporal lavage and sympathomimetic injections at the initial onset, with the rest managed with cytoreductive measures initially. Follow-up data revealed the death of three children, whereas, of those that survived, fourteen had preserved erectile functions after a variable period of time.

**Conclusion:**

Priapism in children warrants a thorough physical examination focusing on organomegaly and a complete hemogram. Initial management should be two-pronged with a priapism-directed corporal-lavage approach and cytoreductive measures for better long-term outcomes.

## Introduction

Priapism refers to the prolonged and persistent erection of the penis, hours beyond (>4 hours) or unrelated to sexual stimulation [1,2]. This persistent erection is due to persistently engorged corpus cavernosum secondary to a vascular disturbance that typically controls penile rigidity. Sickle cell disease is the most common cause of priapism in children (65%), whereas leukaemia-associated priapism in children (LAPC) incidence rates are 10% [3]. Data on LAPC are sparse, and so are the outcomes of its management.

The present review is undertaken to (a) assess the characteristics and types of leukaemia and their role in LAPC, (b) describe the management options available and (c) investigate the outcomes.

## Methodology

**Figure 1.**
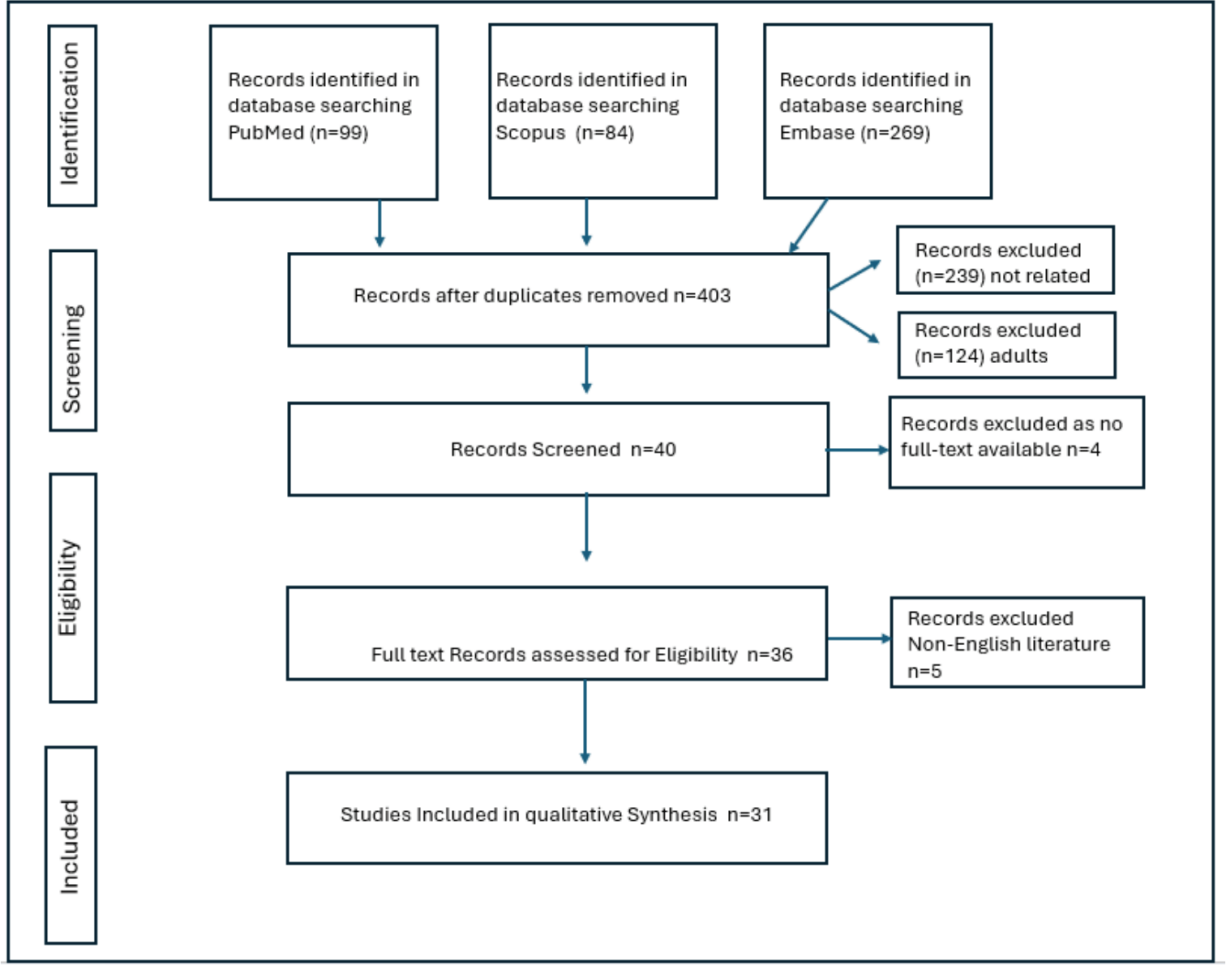
The PRISMA flow diagram detailing the search of articles involving children with leukaemia-associated priapism.

We researched English literature from January 1990 to 2024 (PubMed, Google Scholar, Cochrane, Scopus and Embase) for original articles, reviews, case series and case reports for search terms ‘Priapism’ and (‘Leukaemia’ or ‘Lymphoma’) and (‘childhood’ or ‘all children (birth-18 years)’), excluding terms ‘sickle cell’, ‘female’ and ‘Adult’. We also excluded grey literature data from this review. Thirty-one studies were finally included in our review after excluding duplicates, including those with no full text, and applying our limiters (Figure 1). Our study was registered with PROSPERO ‘CRD42024519022’, and PRISMA-ScR reporting guidelines [4] were adhered to.

## Results

We reviewed a total of 51 cases in 31 different studies. The overall data on LAPC are quite scarce, and this is noted in numerous case reports (28 out of 31 studies). The remaining three studies are case series (≥3 cases), which include a population-based registry study on chronic myeloid leukaemia (CML) and another paper studying the impact of leukapheresis in acute myeloid leukaemia (AML) [5–7]. One case report mentions two cases, one of which was of an adult. Hence, only the paediatric case has been included [8].

The median age in our review was 12 years (3–17 years), and the median duration, in 37.3% of cases after the onset of priapism to presentation in the hospital, was 24 hours (*n* = 19). However, various reports mention recurring or stuttering priapism occurring a few days or a few weeks before the presentation, with no mention of the exact time of presentation from the latest episode of priapism [5, 9, 10]

The search showed the most frequent disorder being CML, 68.9% (*n* = 35). The chronic phase of CML was the most common phase of onset of priapism and presentation to the clinician (71.4%), followed by blast crisis and accelerated phase (11.4% and 8.6%, respectively). The four remaining reports did not mention the blast percentages or the phase of CML. The median age of the CML cohort was 14 years (4–17 years) Of the 35 cases, 32 cases had palpable splenomegaly, and three reports failed to mention the examination findings. The median white blood cell (WBC) count was 450,000/μL (135,000/μL–899,000/μL). Table 1 reviews chronic leukaemia cases and their characteristics, and Table 2 reviews the priapism-directed management and eventual outcomes. Apart from imatinib, hydroxyurea, allopurinol and steroids were recorded medications in these case reports. Thirty-seven percent of cases (*n* = 13) report using corporal lavage with saline or phenylephrine injections and adequate hydration as the initial management step. Surgical intervention in the form of a distal shunt (Winter and Ebbehoj) was performed in six cases, of which one needed revision to a proximal shunt procedure for eventual detumescence [11]. One case documented bilateral internal iliac artery embolization [12]. Very few of these studies mentioned the eventual follow-up outcomes. Of those, three cases of erectile dysfunction [12–14] were mentioned, along with one death secondary to pneumonitis post-stem cell transplant [5].

Of the acute leukaemias (Table 3) associated with priapism, the median age was younger (9.3 years and 3–16 years), and the most common subtype was B cell acute lymphoblastic leukaemia (ALL) followed by T cell ALL and AML. Of the 16 cases of acute leukaemias, 11.8% (*n* = 6) were B-ALL cases, 9.8% (*n* = 5) cases were T-ALL, 5.9% (*n* = 3) AML and 2 cases were ALL with subtype not mentioned. The median WBC in acute leukaemia cases noted was 423,500/μL (27,300/μL–693,000/μL). Acute leukaemias were overall associated with a rapid progression of the underlying disease with an earlier presentation (<24 hours in 43.8% of acute cases, *n* = 7) and two reports of death due to central nervous system (CNS) leukaemia [15] and septic shock [16]. From the available data, 7 cases (43.8%) of acute leukaemias were in remission. However, the overall long-term erectile function outcomes in this cohort are not available (except in one case where the erectile function was preserved [6]).

## Discussion

### Etiopathogenesis

Hinman [17] first reported the ischemic nature of priapism based on aspiration of deoxygenated blood due to increased viscosity, congestion and reduced flow. Eponymous with the Greek–Roman mythological figure Priapus, son of Aphrodite, priapism was first described in the literature in 1616 by Petraens. It was almost 260 years later; this condition was first recorded in a child [18, 19]

Priapism is subdivided into three types: high-flow/non-ischemic/arterial type, low-flow/ischemic type and stuttering/recurring type (common in sickle cell disease). It is the low-flow/ischemic type that is painful, may result in irreversible damage through fibrosis if left untreated for 24–36 hours and has been strongly implicated with leading to impotence in 35%–90% of men [20–24]. It becomes essential to differentiate the clinical type of priapism to prevent long-term, irreversible effects [1, 25–27].

Priapism in leukaemia is hypothesised to occur via different mechanisms [28–30]. The most likely mechanism could occur due to venous obstruction secondary to microemboli/thrombi and increased microviscosity due to increased circulating blood cells (‘symptomatic hyperleukocytosis’, also known as ‘leukostasis’). Hyperleukocytosis is defined as a WBC greater than 1,00,000/μL in patients affected by leukaemias. The incidence of hyperleukocytosis ranges from 5% to 13% in AML and from 10% to 30% in ALL [31]. However, symptomatic hyperleukocytosis is higher in AML than in ALL [32] and also typically occurs with much higher WBC counts in the case of ALL than in patients with AML. The reason is that myeloid blasts are larger and more rigid than lymphoid blasts, and myeloid blasts secrete cytokines, which upregulate endothelial cell adhesion, whereas in chronic leukaemia such as CML, WBCs are usually segmented neutrophils, metamyelocytes and myelocytes, which are smaller and more deformable. Hence, symptomatic hyperleukocytosis is very rare in this patient population and is mostly seen in the accelerated phase or blast crisis or the overall leukocyte counts are extremely high for the symptoms of LAPC to occur [29, 30, 33, 34].

In addition, increased mechanical pressure from veins draining the spleen, which is often enlarged in paediatric CML, and infiltration of the nervous system by leukemic cells, more so in acute leukaemias, have also been implicated in causing priapism [5]. Microscopically, the release of cytokines by malignant cells causes endothelial cell activation, which further causes increased sequestration of leukemic cells in the microvasculature. One report also suggests the isolation of leukemic cells that directly infiltrated into the penile tissue, leading to penile mass in AML [35], while another suggests an involvement of the hypothalamus in LAPC [16]

### Incidence and clinical presentation

CML is a myeloproliferative neoplasm of mature granulocytes. Three stages of CML exist: chronic phase, accelerated phase and blast phase. In one of the largest paediatric cohort studies on priapism in children due to CML, Suttorp *et al* [5] reported an incidence of priapism of 3.2%, with 75% in the chronic phase of CML. This is similar to the findings of our review (71.5%). Ninety percent of paediatric cases of CML are discovered during the chronic phase.

Acute leukaemias with priapism are an even rarer finding. During the initial presentation, some report concomitant CNS symptoms such as headaches and blurred vision with retinal bleeding [15, 36–38], while some report fatigability owing to anaemia, petechiae and rashes, renal insufficiency owing to tumour lysis syndrome, neck swelling and lower urinary tract symptoms [6, 10, 16, 37, 39]. The underlying disease progress is much more rapid in ALL patients, and hence, the incidence of LAPC recorded is much rarer in this subgroup of patients. Three cases are reported with AML, of which Salou *et al* [9] report the priapism associated with the deposition of leukemic cells in extramedullary sites leading to myeloid sarcoma of the sacral canal. Priapism is reported during treatment in this report; however, this finding was not substantiated in another study on conus medullaris syndrome due to myeloid sarcoma leading to urinary and defecation disorders [40].

### Management and long-term outcomes

Priapism is a urological emergency that needs to be addressed early to prevent permanent risk of erectile dysfunction, which is predicted to be >90% if it lasts for more than 24 hours [41]. Children with underlying leukaemia need to be started on disease-directed therapy at the earliest, be it chemotherapy, tyrosine kinase inhibitor or cytoreductive measures with hydration, hydroxyurea and leukapheresis when warranted. However, these measures should not defer the emergency treatment of priapism itself.

**Figure 2.**
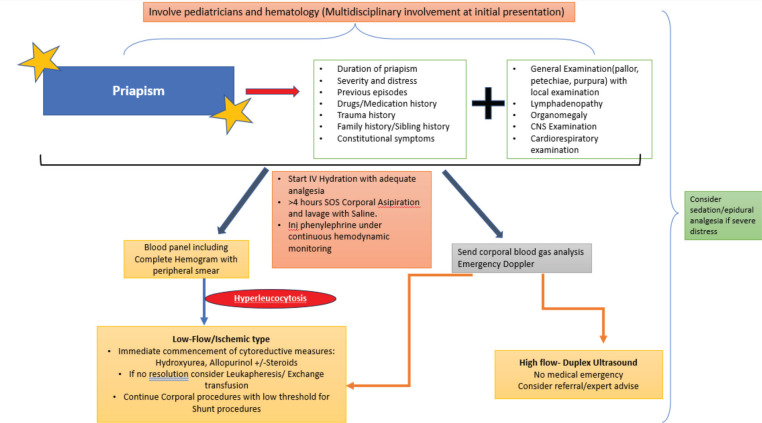
Algorithm for management of LAPC.

Figure 2 depicts a proposed management algorithm for patients presenting with LAPC. The initial management is crucial as it dictates the erectile outcomes and long-term prognosis of these patients. In our study, 20 cases (39.2%) were reportedly subjected to corporal lavage in addition to disease-directed measures. The rest were managed with cytoreductive measures supplemented with adequate IV hydration and analgesia.

Systemic interventions, such as cytoreductive measures with hydroxyurea, allopurinol and steroids, have been shown to reduce the white cell count [5, 42]. In cases where an acute control of leucocyte count is warranted or the cytoreductive measures fail to establish a reduced leucocyte count, leukapheresis or exchange transfusion methods are commonly employed [5, 43, 44]. Seventeen patients (33%) underwent leukapheresis, of which 12 were noted in CML cases. Suttorp *et al* [5], Ponniah *et al* [43] and Veljković *et al* [45] all demonstrate its usefulness for CML cases associated with priapism.

Certain adjunct treatments noted in our review worth revisiting were bilateral internal iliac artery embolization with urokinase [12] and epidural analgesia [37]. If a paediatric anaesthetist is present, low-dose ketamine has been suggested to resolve priapism in some cases and may help to relieve pain during corporal lavage and shunt procedures in these children [46–50]

From a surgical standpoint, a urologist must be aware of various types of shunt procedures that may be performed in cases of unresolved priapism. Distal shunts, such as the Winter, Ebbehoj and T-Shunt, are easier to create. Seideman and Gitlin [51] describe the successful use of a T-Shunt in refractory ischemic priapism in a paediatric patient. In this review, we report a similar use of distal shunts in refractory priapism [5, 14, 52–55]. Thakur *et al* [11] report failure of distal shunt, which was later converted to a proximal shunt with success.

Fifteen cases (29.4%) from the entire cohort reported a preserved erectile function on follow-up. Follow-up data are as sparse as the original literature on LAPC. However, it is of utmost importance that on the initial presentation itself, the patient’s family must be made aware of the possible loss of erectile function in the future, despite the clinician’s best efforts. Teenagers and their parents often do not discuss sexual health in boys [26, 42]. This was also witnessed in a cohort of boys with sickle cell disease with priapism, who reported embarrassment to report their complaints to their parents, even in the emergency room when it became a medical emergency [27].

There are definite limitations present in this review, the first of which is the prevalence of only case reports, as this condition is rare. This gives rise to varied management methods, which need streamlining, as we attempted in our study. Follow-up data on many children in terms of erectile status remain unknown. A multi-institutional collaboration with their respective registries may further help define a protocol to manage LAPC and help to trace and contact the patient or next of kin and provide follow-up data.

## Conclusion

Though rare, priapism may be the only presenting symptom of haematological malignancies in children. Thus, it is essential to examine for organomegaly and do a complete hemogram with peripheral smear examination in all children presenting with priapism. Considering ischemic priapism, a urological emergency and the potential risk of permanent damage later in life, managing solely with systemic anti-leukaemia therapies (chemotherapy and/or leucopheresis) may not be sufficient for early resolution. Concurrent cavernosal management with corporal lavage and injectable sympathomimetics may help resolve the acute condition and prevent long-term erectile sequelae and psychosocial problems.

## Conflicts of interest

The authors declare that they have no known competing financial interests or personal relationships that could have appeared to influence the work reported in this paper.

## Funding

This research did not receive any specific grant funding agencies in the public, commercial or not-for-profit sectors.

## Figures and Tables

**Table 1.** Characteristics of LAPC with CML.

Reference	Case No.	Age	Type of leukaemia	Onset to presentation (duration of priapism)	Blood panel			Splenomegaly	Phase	Blast %
					Haemoglobin (g%)	Platelet count (×10^5^/μL)	WBC (/μL)			
[56]	1	14	CML	24 hours	9.9	3.1	226,900	Yes	Chronic	10
[55]	2	16	CML	11 days	5.7	7.1	614,800	Yes	Blast	60
[57]	3	12	CML	Stuttering type (Intermittent 2 weeks)	9	9.2	346,000	Yes	Chronic	2
[10]	4	7	CML	48 hours	8.84	2.8	400,000	Yes	NA	NA
[58]	5	15	CML	Stuttering type (two months)	9.4	1.3	480,000	Yes	Chronic	2
[59]	6	12	CML	2 days	8.2	3.5	460,000	Yes	NA	NA
[6]	7	9	CML	-	10.1	1.2	509,000	Yes	Chronic	NA
[6]	8	9	CML	Stuttering type (4 days)	10.3	6.6	169,000	Yes	Chronic	NA
[6]	9	9	CML	9 hours	8.2	12.4	472,000	Yes	Blast	NA
[14]	10	16	CML	Intermittent 1 month	9.9	5.7	542,700	Yes	Chronic	0
[12]	11	14	CML	24 hours	6.5	6.4	510,000	NA	NA	NA
[52]	12	13	CML	24 hours	8.5	4.5	350,000	Yes	Chronic	2
[45]	13	16	CML	24 hours	11	4.2	320,000	Yes	Chronic	3
[13]	14	14	CML	Stuttering (2 months)	11.2	3.2	458,000	Yes	Chronic	5
[53]	15	8	CML	3 days	9.7	7.1	285,000	Yes	Chronic	2
[60]	16	17	CML	2 days	9.2	2.7	386,000	Yes	Chronic	7
[61]	17	11	CML	12 hours	NA	5.5	290,000	Yes	Chronic	NA
[11]	18	15	CML	2 days	9	2.0	135,000	Yes	Chronic	3
[62]	19	17	CML	24 hours	11.3	7.3	377,000	Yes	Accelerated	12
[5]	20	4	CML	24 hours	5.1	0.7	899,000	Yes	Blast	NA
[5]	21	7	CML	<12 hours	8.4	5.4	508,000	Yes	Chronic	NA
[5]	22	7	CML	>6 weeks, stuttering	4.9	1.9	635,000	Yes	Chronic	NA
[5]	23	9	CML	5–7 days stuttering	9.2	4.0	483,000	Yes	Accelerated	NA
[5]	24	10	CML	11 hours	6.4	3.6	629,000	Yes	Chronic	NA
[5]	25	11	CML	NA	7.6	6.6	459,000	Yes	Chronic	NA
[5]	26	11	CML	>12 hours	7.1	8.3	368,510	Yes	Chronic	NA
[5]	27	13	CML	Several days stuttering	6.7	11.3	339,000	Yes	Chronic	NA
[5]	28	14	CML	Several days stuttering	8	3.7	407,840	Yes	Chronic	NA
[5]	29	14	CML	>3 months, stuttering	9.1	2.7	450,000	Yes	Chronic	NA
[5]	30	15	CML	NA	6.5	4.3	657,270	Yes	Accelerated	NA
[5]	31	15	CML	11 hours	8.5	9.8	350,240	Yes	Chronic	NA
[5]	32	16	CML	<2 weeks stuttering	8.1	2.5	481,170	Yes	Chronic	NA
[5]	33	17	CML	NA	9.7	3.9	236,700	Yes	Chronic	NA
[5]	34	17	CML	NA	14	4.3	355,470	Yes	Chronic	NA
[5]	35	17	CML	NA	9.8	1.5	481,000	Yes	Blast	NA

**Table 2.** Management and outcomes of LAPC with CML.

Reference	Case No.	Priapism directed Mx	Leukapheresis	Oncological Mx of CML	Surgical approach for priapism	Time to final resolution of priapism	Erectile function status	Long-term outcome (Follow-up duration)
[56]	1	Corporal lavage with cytoreductive measures	No	Cytoreductive (HU and allopurinol)l	Corporal lavage and phenylephrine injection	5 days	Preserved erectile function	Remission (2 month)
[55]	2	Shunt procedure with cytoreductive measures and blood transfusion	No	Cytoreductive (HU, aspirin and allopurinol)	Corporal lavage and winter shunt creation	13 days	Preserved erectile function	NA
[57]	3	Subcutaneous Terbutaline with cytoreductive measures	No	Imatinib with cytoreductive (HU and allopurinol)	No	48 hours	NA	NA
[10]	4	High-dose methylprednisolone (HDMP) and busulphan	No	HDMP with busulphan with tapering of steroids	No	5 days	NA	Remission (18 months)
[58]	5	Cytoreductive measures	Yes	HU and aSCT	No	NA	NA	NA
[59]	6	Busulphan, cytoreductive measures and blood transfusion	No	Busulphan and cytoreductive measures	No	12 days	NA	Na
[6]	7	Cytoreductive measures and LMWH	Yes	HU and Cyclophosphamide	No	30 days	Preserved erectile function	NA
[6]	8	Cytoreductive measures and LMWH	No	HU	No	3 months	Preserved erectile function	NA
[6]	9	Cytoreductive measures and LMWH	Yes	Cyclophosphamide	No	20 days	Preserved erectile function	NA
[14]	10	Cytarabine, cytoreductive measures and corporal shunt	No	Imatinib, cytarabine, HU and allopurinol	Ebbehoj shunt	22 days	Erectile dysfunction	Remission (10 months)
[12]	11	Cytoreductive measures, urokinase and embolization	No	HU and injection urokinase	Bilateral internal iliac artery embolization	4 days	Erectile dysfunction	NA
[52]	12	Cytoreductive measures, phenylephrine and distal shunt	Yes	Imatinib, HU and allopurinol	Corporal lavage, inj phenylephrine followed by distal shunt	5 days	NA	NA
[45]	13	Cytoreductive measures	Yes	HU and imatinib	No	13 days	NA	NA
[13]	14	Cytoreductive and surgical decompression	Yes	HU, allopurinol and imatinib	Corporal lavage followed by bilateral cavernotomy	8 days	Erectile dysfunction	Remission (3 months)
[53]	15	Phenylephrine inj and corporal shunt	No	Dasatinib	Distal shunt after corporal lavage	NA	NA	Remission (2 months)
[60]	16	Corporal lavage, phenylephrine and cytoreductive measures	No	HU and imatinib	Corporal lavage	8 days	Preserved erectile function	Remission (2 years)
[61]	17	Cytoreductive measures	No	HU and allopurinol	No	24 hours	Preserved erectile function	Remission (duration unknown)
[11]	18	Corporal shunt with cytoreductive measures	No	HU and imatinib	Distal shunt followed by proximal corporospongiosal shunt	4 days	NA	Remission (2 years)
[62]	19	Corporal lavage and inj phenylephrine injection	No	None	Corporal lavage and inj phenylephrine injection	24 hours	NA	NA
[5]	20	Exchange transfusion	No	Imatinib, stem cell transplant allogenic	No	NA	Preserved erectile function	Remission
[5]	21	Leukapheresis, corporal lavage, adrenaline injection and corporal shunt	Yes	Imatinib	Corporal lavage, adrenaline injection and corporospongiosal shunt	NA	NA	Remission
[5]	22	Corporal lavage, leukapheresis and cytarabine	Yes	Imatinib and cytarabine	Corporal lavage	NA	Preserved erectile function	Remission
[5]	23	Cytoreductive measures	No	Imatinib and HU	No	NA	Preserved erectile function	Remission
[5]	24	Leukapheresis, cytoreductive measure and cytarabine	Yes	Imatinib, cytarabine and HU	No	NA	Preserved erectile function	Remission
[5]	25	NA	No	Imatinib and HU	NA	NA	NA	Remission
[5]	26	NA	No	Imatinib	NA	NA	NA	Remission
[5]	27	Corporal lavage, inj adrenaline, LMWH, urokinase and cytoreductive measure	No	HU, aSCT and chemotherapy (FRALLE 2000)	Corporal lavage	NA	Early death	Death: Pneumonitis+ Sepsis
[5]	28	Cytoreductive measures, corporal lavage, inj adrenaline and leukapheresis,	Yes	Imatinib and HU	Corporal lavage	NA	NA	Remission
[5]	29	Exchange transfusion, cytarabine	No	Imatinib, exchange transfusion and cytarabine	No	NA	Preserved erectile function	Remission
[5]	30	NA	No	Imatinib, interferon and aSCT	No	NA	NA	Remission
[5]	31	Corporal lavage, inj adrenaline, leukapheresis and cytoreductive measure	Yes	Imatinib with dasatinib switch, HU	Corporal lavage	NA	Preserved erectile function	Remission
[5]	32	Corporal lavage, leukapheresis and cytoreductive measures	Yes	HU and imatinib	Corporal lavage	NA	Preserved erectile function	Death: CNS bleed
[5]	33	NA	NA	Imatinib	No	NA	NA	Remission
[5]	34	NA	NA	Imatinib	No	NA	NA	Remission
[5]	35	Heparin and prednisolone	No	Imatinib and aSCT	No	NA	NA	Remission

Hu- Hydroxyurea

HDMP- High-dose methylprednisone

LMWH- Low molecular weight heparin

aSCT- Allogenic stem cell transplant

FRALLE 2000-French Association of Pediatric Haematology and Oncology protocol for ALL

NA- No available data

**Table 3.** Characteristics, management and outcome of LAPC with acute leukaemia.

Ref	Casenumber	Age	Type of leukaemia	Duration of priapism at onset of treatment	Blood panel			Splenomegaly	Blast (%)	Management			Erectile function	Prognosis
					Hb	WBC (/μL)	Platelet (×10^5^/μL)			Oncologic Rx	Priapism directed manoeuvres	Leukapheresis		
[38]	36	15	T-ALL	6 hours	NA	600,000	6.6	No	97	Steroids, cytoreductive measure and chemotherapy	No	No	NA	Remission (Day 22)
[36]	37	12	B-ALL	2 days	6.2	501,000	0.76	No	95	NA	NA	NA	NA	NA
[15]	38	10	B-ALL	NA	5	693,000	0.40	No	95	Cytoreductive therapy (allopurinol) and steroids	Corporal lavage and inj phenylephrine	No	Death	Death after 48 hours: CNS leukaemia with respiratory failure
[8]	39	9	AML	3 days	3.4	82,000	0.81	Yes	92	NA	Corporal lavage, inj phenylephrine and induction chemotherapy Vincristine+ Prednisolone	No	NA	NA
[63]	40	9	B-ALL	18 hours	9.3	583,000	0.51	No	90	Cytoreductive therapy and chemotherapy as per ALL IC BFM 2009	Corporal lavage	Yes	NA	Remission (Duration?)
[9]	41	6	AML with sacral myeloid sarcoma	NA	9.5	27,300	1.80	No	28	AML-BFM-2012 protocol Chemotherapy and aSCT	Pelvic floor exercise	No	NA	Remission (2 months)
[10]	42	8	B-ALL	24 hours	7.0	537,000	0.40	Yes	100	HDMP and cytoreductive therapy	No	No	NA	Remission (7 months)
[16]	43	6	ALL (Type not mentioned)	NA	9.0	270,000	0.40	Yes	95	MTX, cyclophosphamide, vincristine and HDMP	No	No	NA	Death: Bone marrow aplasia with sepsis
[64]	44	3	T-ALL	>3hours	7.0	606,300	0.20	Yes	97	Vincristine, daunorubicin, asparginase and prednisolone	Corporal lavage and inj epinephrine	Yes	NA	Remission (8 months)
[6]	45	13	T-ALL	10 hours	14	274,000	1.20	Yes	NA	Steroids	No	No	Preserved function	Remission (4-8 years)
[65]	46	12	ALL (Type not mentioned)	NA	NA	618,000	NA	NA	100	Steroids and cytoreductive therapy	No	Yes	NA	NA
[37]	47	16	T-ALL	12 hours	NA	271,000	NA	NA	97	Steroids and chemotherapy as per ALL IC BFM 2009	Corporal lavage followed by epidural analgesia	No	NA	NA
[54]	48	14	AML	24 hours	8.5	346,900	Low	NA	96	Cytoreductive therapy-HU	Corporal lavage and inj phenylephrine	No	NA	NA
[39]	49	7	T-ALL	NA	11.4	298,000	0.62	Yes	82	Cytoreductive therapy and chemotherapy as per Children’s Oncology Group Trial AALL1231 protocol	Corporal lavage	Exchange transfusion	NA	Remission (Duration?)
[66]	50	5	B-ALL	NA	6.5	453,000	0.55	Yes	91	Chemotherapy, cytoreductive therapy and steroids	No	No	NA	NA
[7]	51	3	B-ALL			394,000				Steroids	No	Yes	NA	NA
